# Geographical distribution of pyrethroid resistance mutations in *Varroa destructor* across Türkiye and a European overview

**DOI:** 10.1007/s10493-023-00879-z

**Published:** 2024-02-24

**Authors:** Esengül Erdem, Nafiye Koç-İnak, Mustafa Rüstemoğlu, Emre İnak

**Affiliations:** 1 Plant Protection Department, Faculty of Agriculture, Şırnak University, Şirnak, Turkey; 2 Department of Parasitology, Faculty of Veterinary Medicine, Ankara University, Altindag, 06070 Ankara, Turkey; 3 Department of Plant Protection, Faculty of Agriculture, Ankara University, 06110 Ankara, Turkey

**Keywords:** Target-site mutations, Honey bee, Pyrethroid resistance, *Varroa destructor*

## Abstract

*Varroa destructor* Anderson & Trueman (Acari: Varroidae) is of paramount significance in modern beekeeping, with infestations presenting a primary challenge that directly influences colony health, productivity, and overall apicultural sustainability. In order to control this mite, many beekeepers rely on a limited number of approved synthetic acaricides, including the pyrethroids tau-fluvalinate, flumethrin and organophosphate coumaphos. However, the excessive use of these substances has led to the widespread development of resistance in various beekeeping areas globally. In the present study, the occurrence of resistance mutations in the voltage-gated sodium channel (VGSC) and acetylcholinesterase (AChE), the target-site of pyrethroids and coumaphos, respectively, was examined in *Varroa* populations collected throughout the southeastern and eastern Anatolia regions of Türkiye. All *Varroa* samples belonged to the Korean haplotype, and a very low genetic distance was observed based on cytochrome c oxidase subunit I (*COI*) gene sequences. No amino acid substitutions were determined at the key residues of AChE. On the other hand, three amino acid substitutions, (L925V/I/M), previously associated with pyrethroid resistance, were identified in nearly 80% of the Turkish populations. Importantly, L925M, the dominant mutation in the USA, was detected in Turkish *Varroa* populations for the first time. To gain a more comprehensive perspective, we conducted a systematic analysis of the distribution of pyrethroid resistance mutations across Europe, based on the previously reported data. *Varroa* populations from Mediterranean countries such as Türkiye, Spain, and Greece exhibited the highest frequency of resistance mutation. Revealing the occurrence and geographical distribution of pyrethroid resistance mutations in *V. destructor* populations across the country will enhance the development of more efficient strategies for mite management.

**Supplementary Information:**

The online version contains supplementary material available at 10.1007/s10493-023-00879-z.

## Introduction

The ectoparasitic honey bee mite *Varroa destructor* Anderson & Trueman (Acari: Varroidae) is a destructive parasite of the Western honey bee *Apis mellifera* and is widely recognized to be one of the primary reason for colony collapse disorder (CCD) (Rosenkranz et al. 2010a, b; Le Conte et al. 2010). In addition to directly feeding on the fat body and hemolymph of bees (Ramsey et al. 2019), *Varroa* mites can transmit several virus diseases, including Deformed Wing Virus (DWV), which can be lethal to honey bee colonies (McMenamin and Genersch 2015; Martin and Brettell 2019). Although organic acids have been increasingly used by a growing number of beekeepers, the main control of *Varroa* has still largely relied on chemical acaricides worldwide (Rosenkranz et al. 2010a, b; Tutun et al. 2018; Gregorc and Sampson 2019; Brodschneider et al. 2023). It has long been a critical issue to kill mites in-hive without any damage to bees. The acaricides such as tau-fluvalinate, flumethrin, amitraz, and coumaphos are known to offer such selectivity and thus have been used for decades in *Varroa* control (van der Steen and Vejsnæs, 2021). In the case of pyrethroids, the amino acid variations in the voltage-gated sodium channel (VGSC), leading to differential acaricide binding, have been suggested as the primary factor underlying the selectivity between *Varroa* and *A. mellifera*, as well as the common eastern bumblebee *Bombus impatiens* (O’Reilly et al. 2014; Wu et al. 2017). Repetitive selection, driven by the limited number of available acaricides in the portfolio, has inevitably resulted in resistance development in *Varroa* populations (Elzen et al. 1999; Spreafico et al. 2001; Martin 2004; Pettis 2004; Mitton et al. 2022). Due to honey bee trade, migratory beekeeping, and the various dispersal strategies of *Varroa* mites (Sammataro et al. 2000; Rosenkranz et al. 2010a, b; Jack and Ellis 2021), resistance can quickly spread over wide areas once it arises, making the issue even more challenging to address.

Resistance development in *Varroa* mites has been mainly associated with increased detoxification enzyme activity and a number of point mutations in the target-site of pyrethroids (Van Leeuwen and Dermauw 2016; Mitton et al. 2022; De Rouck et al. 2023). Current knowledge of metabolic aspects of pyrethroid resistance mechanisms is primarily based on biochemical and synergism assays (Hillesheim et al. 1996; Mozes-Koch et al. 2000), with relatively limited information available regarding specific mechanisms. Development of metabolic resistance can be complex in *Varroa*, as recently demonstrated in the case of coumaphos resistance, which results from the downregulation of a P450 enzyme, leading to decreased activation (Vlogiannitis et al. 2021a). On the other hand, no mutation conferring resistance to coumaphos in *Varroa* mites has been reported so far, although target-site mutations are known as a common resistance mechanism for other acetylcholinesterase inhibitors (Feyereisen et al. 2015).

Point mutations in the VGSC associated with tau-fluvalinate resistance in *V. destructor* were first uncovered in the USA (Wang et al. 2002). Specifically, the L1596P (*Musca domestica* numbering) mutation in the linker connecting domains III and IV in *V. destructor* was further examined since the proline (P) is conserved in insects and had a potential to be one of the reasons for low toxicity of fluvalinate to bees. Indeed, functional validation in *Xenopus* oocytes, using a cockroach sodium channel, showed a fivefold increase in sensitivity to fluvalinate when proline was replaced with leucine (L) (Liu et al. 2006). However, this mutation was not detected anymore in following studies. Years later, a L925V mutation in the transmembrane segment 5 of domain II of the VGSC was uncovered in *V. destructor* populations from the UK and Czechia (González-Cabrera et al. 2013; Hubert et al. 2014). Today, the presence of the L925V mutation has been reported in other countries and continents (Alissandrakis et al. 2017; Panini et al. 2019; Stara et al. 2019; Koç et al. 2021; Vlogiannitis et al. 2021b; Mitton et al. 2021; Ogihara et al. 2021; Hernández-Rodríguez et al. 2021; Millán-Leiva et al. 2021a). Therefore, similar to other arthropods, resistance to pyrethroids in European populations of *V. destructor* is thought to be primarily driven by the target-site mutation L925V. (González-Cabrera et al. 2018). Other mutations in the same position (L925I and L925M) have also been documented, especially in *Varroa* samples from the USA (González-Cabrera et al. 2016). More recently, the *knockdown resistance-like* (*kdr-like*) mutation M918L in the IIS4—S5 channel linker, a variant of the well-described *super-kdr* mutation (M918T), in combination with L925V was detected in *V. destructor* populations from Eastern Spain (Millán-Leiva et al. 2021b). The M918L together with other mutations was also found in insect and other mite species (Carletto et al. 2010; Karatolos et al. 2012; Katsavou et al. 2020). The role of both L925I and M918T (*super-kdr*) in pyrethroid resistance was functionally validated by electrophysiology and molecular docking analyses (Vais et al. 2003; Usherwood et al. 2007).

Türkiye is the second largest honey-producing country following China (FAOSTAT 2023). However, the expected honey production yield has not been achieved due to several factors. Among these, *Varroa* mites and their ability to develop resistance, resulting in failure in chemical control, are regarded as a primary and significant threat. Koç et al. (2021) documented the widespread distribution of the L925V/I mutations in *Varroa* populations from the central Anatolia region. This finding highlights the significance of conducting resistance monitoring in additional geographic areas, such as the eastern Anatolia region, where traditional beekeeping is widespread. Therefore, in the present study, we screened well-known pyrethroid resistance mutations, including L925V/L/M, along with the *super-kdr* M918T mutation. We also investigated mutations at key positions in the target site of coumaphos, acetylcholinesterase (AChE), in *Varroa* populations. Next, we reviewed the occurrence and distribution of pyrethroid resistance mutation across the country, as well as in Europe. Last, a haplotype network analysis was performed to analyze the relationship among worldwide *Varroa* populations.

## Materials and methods

### Origin of Varroa destructor populations

A total of 44 *Varroa* populations, each from a different apiary, were sampled across 21 cities in southeastern and eastern Anatolia regions of Türkiye during 2022 (Fig. 2B and Table S1). Mites were collected with the powdered sugar method as outlined by Gregorc et al. (2017) and subsequently transferred to the laboratory in 90% ethanol for molecular analysis. Further information regarding the history of pesticide usage, beekeeping practices, and the location of the apiaries are presented in Table S1.

### Genomic DNA extraction

The genomic DNA (gDNA) was extracted from pools of 10 adult females per population, using the DNeasy Blood & Tissue Kit (Qiagen, Germany) according to the manufacturer's instructions. In the final stage, DNAs were eluted using 100 μl of elution buffer. The quality and quantity of the gDNA were assessed through agarose gel electrophoresis (1.5%) and a spectrophotometer (NanoDrop™ 2000, Thermo Scientific, USA). The DNA samples were then stored at − 20 °C until needed.

### Amplification of target-sites and mtDNAs

The presence of the resistance mutations located at the IIS4–IIS5 region of the VGSC gene was screened as previously described (Alissandrakis et al. 2017). New primers were designed for VdAChE1 (accession number: XP_022645444), the major catalytic enzyme in *V. destructor* (Kim et al. 2022), using primer3 (Untergasser et al. 2007). These primers allow screening for key residues previously associated with resistance to AChE inhibitors. The primers were as follows: VdAChE1_F1: ATACGCTAAACCGCCGATTG; VdAChE1_R1: TCGTCACATTGTTGGGGTTG and VdAChE1_F2: CCGTTAAGTGCCTTCGACAG; VdAChE1_R2: CTCGACAAAGTCCTCTCGGG (see Fig. S1 for amplified fragments). The *Varroa* samples (n = 22) collected by Koç et al. (2021) was also included for the screening of AChE mutations. PCR condition for AChE primers were as follows: 5 min at 95 °C, 35 cycles of 30 s at 95 °C, 30 s at 53 °C and 30 s at 72 °C and a final extension of 5 min at 72 °C.

The genetic distance between *Varroa* mite populations from different locations was determined by analyzing the mtDNA sequences of the cytochrome c oxidase subunit I (*COI*) (10KbCOIF1-6,5KbCOIR primers) and NADH dehydrogenase subunit 4 (*ND4*) (ND4F- ND4R primers) genes that were amplified according to Navajas et al. (2010) and Muntaabski et al. (2020).

Each PCR reaction was performed with Promega GoTaq® Flexi kit in a final volume of 50 μL consisting of 2 μL of mite DNA (ranging from 40 to 65 ng/μL), 3 µL of MgCl, 1 µL of dNTP, 10 µL of 5X Buffer, 2.5 µL of each of forward and reverse primer, and 0.25 µL Taq DNA polymerase. The amplified products were visualized on a 1.5% agarose gel stained with ethidium bromide using a UV transilluminator. PCR amplicons were purified with the EZNA Cycle-Pure kit (Omega Biotek, USA) and subsequently sequenced at LGC Genomics (Berlin, Germany).

### Data analysis and haplotype network

The presence and frequency of pyrethroid mutations, both in this study and in our previous study (Koç et al. 2021), were evaluated by analyzing the peak heights in sequencing chromatographs, following the methodology described by Van Leeuwen et al. (2008). The maps were generated using mapchart.net with inputs from current and previous studies (see Table S2-S3 for details on the data used and the corresponding references). All previous studies employed individual mites for pyrethroid mutation identification, with the exception of Farjamfar et al. (2018), who used pooled DNA but found no mutations in their study. Therefore, only homozygous mutant individuals were considered resistant when generating the map, given the recessive nature of the pyrethroid resistance mutation (González-Cabrera et al. 2013, 2018).

To explore the genetic diversity among various *Varroa* populations, we examined the sequences obtained in our study together with sequences retrieved from the GenBank database (see Table S4 for accession numbers of sequences used in analysis). Because we used pooled mites for DNA extraction in this study, we could not determine individual haplotypes. Therefore, we used consensus sequences based on dominant nucleotides as representative haplotypes for each sample. MAFFT v.7 with the ‘Auto’ strategy (Katoh et al. 2019) was utilized to perform a multiple sequence alignment, which was later refined using Bioedit v.7.0.5 software (Hall 1999). Haplotype analysis of *COI* sequences was conducted using DnaSP v6.12.03 (Rozas et al. 2017) and PopArt v1.7 was used to draw haplotype networks based on the haplotypes generated by DnaSP (Leigh and Bryant 2015). The accession numbers of sequences obtained in this study (both *COI* and *ND4*), as well as those retrieved from GenBank for analysis, along with their haplotypes, are provided in Table S2 and Table S4. The genetic distance among all the obtained sequences was assessed using MEGA11 (Tamura et al. 2021).

## Results and discussion

*Varroa destructor* is widely recognized as a significant factor contributing to the decline of honey bee colonies (Traynor et al. 2020). In addition to their considerable economic importance to Türkiye through honey exportation, honey bees play a crucial role in pollinating numerous crops. However, *Varroa* control seems to be not at the intended level, as 100% infection rates have been previously reported in Turkish apiaries (Yalçınkaya and Keskin 2010; Koç et al. 2021). Similarly, all hives in the study's sampling area were infested with *Varroa* mites, albeit with varying levels of severity as observed through visual inspection.

Arrhenotokous reproduction together with the dispersal ability of resistant individuals due to their parasite biology, the honey bee trade, and migratory beekeeping all contribute to the rapid development and spread of resistance across large areas (Carrière 2003; Miller and Sappington 2017; Sammataro et al. 2000). In this study, we conducted a molecular screening for well-known pyrethroid resistance mutations in *Varroa* populations from the southeastern and eastern Anatolian regions of Türkiye. These regions are of significant importance to beekeeping due to being major stops for numerous migratory beekeepers across the country and their diverse and distinct flora, which results in the production of high-quality and multifloral honeys (Akkaya and Alkan 2007; Güler and Demir 2005).

**Fig. 1:**
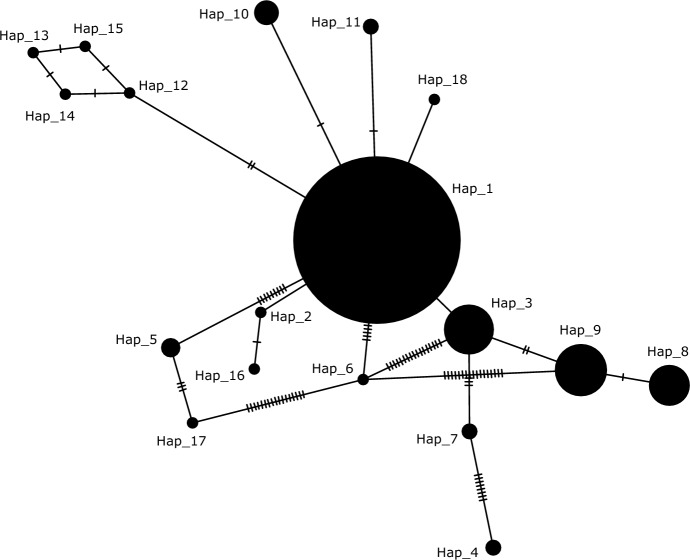
Haplotype network analysis based on COI sequences of worldwide samples of *Varroa destructor*. The sizes of the circles correspond to the frequency of each haplotype, and each solid line represents a mutation step that connects two haplotypes

Two highly virulent haplotypes, namely the Korean and Japanese/Thailand haplotypes, of *V. destructor* shifted their host from the Asian honey bee (*Apis cerana*) to the European honey bee (*Apis mellifera*) in the Far East over fifty years ago, and since then, they have extensively spread across the globe (Traynor et al. 2020). This host shift resulted in a genetic bottleneck, leading to reduced genetic variation within *V. destructor* populations (Solignac et al. 2005; Navajas et al. 2010). The comparison of 332 *COI* sequences analyzed in the present study revealed very high similarity (only 0.06% variation), supporting the evolutionary history of *V. destructor*. Similar to previous findings in Türkiye (Warrit et al. 2004; Ayan and Aldemir 2018; Koç et al. 2021), all *V. destructor* populations investigated in the present study belonged to Korean haplotype. To provide a broader perspective, we conducted a haplotype analysis using over 300 sequences from worldwide populations of *Varroa* mites (Fig. 1). Hap_1 emerged as the most prevalent haplotype, encompassing all Turkish *Varroa* sequences. The haplotype network analysis aligns with the genetic bottleneck hypothesis, primarily attributed to host shifting, as the majority of the haplotypes belong to single haplotype (Hap_1) (Fig. 1). Another mitochondrial gene, *ND4*, has been suggested as a sensitive marker for detecting genetic variations in *V. destructor* (Muntaabski et al. 2020). In our study, we observed no genetic variation (0%) among *ND4* sequences, while a minimal variation of 0.03% was observed in *COI* sequences among Turkish *Varroa* populations.

AChE inhibitors including organophosphates (OPs) and carbamates have been long used to control pests or vector species (Sparks and Nauen 2015), although most of them have been banned today. The first case of coumaphos resistance was reported more than 20 years ago in Italian populations of *Varroa* (Spreafico et al. 2001), and it has since been found on different continents (Pettis 2004; Sammataro et al. 2005; Maggi et al. 2009, 2011; Hernández-Rodríguez et al. 2021). However, the molecular mechanisms underlying the development of resistance remain unknown. A number of target-site mutations have been reported to confer resistance to AChE inhibitors in invertebrates (Lee et al. 2015). Nevertheless, as of now, no such mutations have been reported within *Varroa*. In the present study, a comprehensive molecular screening, encompassing 66 populations across the country, was conducted. In line with previous reports, no mutations were detected at the key residues of AChE. Since we lack information regarding the phenotypic susceptibility levels to coumaphos, it is challenging to make a definitive statement ruling out target-site mutations as a resistance mechanism in Turkish *Varroa* populations. A recent study showed that resistance to coumaphos can indeed arise through mechanisms other than target-site mutation. Vlogiannitis et al. (2021a, b) documented the downregulation of a P450 gene, *CYP4EP4*, which results in reduced activation of coumaphos. More studies are needed to show if this mechanism is shared across *Varroa* populations from geographical backgrounds.

Target-site mutations have been suggested as the primary mechanism responsible for pyrethroid resistance in *V. destructor* (Dong et al. 2014; De Rouck et al. 2023), allowing the implementation of molecular screening for resistance (Van Leeuwen et al. 2020). The results of this study showed that approximately 80% of the sampled *Varroa* populations contained at least one of the mutations (L925V, L925I, or L925M). Specifically, both L925V and L925I mutations appear to be prevalent in the country (Koç et al. 2021; this study). Fifteen out of the 44 samples contained no susceptible wild-type alleles. Among them, five samples exhibited fixation for either L925V or L925I, whereas the rest of the 11 samples had mixed mutations, without any mites having susceptible leucine (L) at position 925. Some beekeepers in the sampled regions have opted to discontinue the application of flumethrin due to the treatment's inefficacy (personal communications). Notably, upon examining the genotyping results, we observed a fixed L925V/I mutation in all areas where flumethrin was not used, highlighting the potential of these mutations as molecular diagnostics for pyrethroid resistance. Conversely, none of the Turkish *Varroa* populations had M918L mutation, in line with Koç et al. (2021). This study, along with Koç et al. (2021), clearly demonstrates the widespread distribution of pyrethroid resistance mutations within the Turkish *Varroa* populations; however, the frequencies of these mutations differed significantly (Table S2). In addition to populations with fixed mutations, populations having a low frequency of homozygous resistant individuals can also be easily selected after repeated rounds of selection. Therefore, they may also present a potential risk for *Varroa* control. Since pooled DNAs were used in both present study and Koç et al. (2021), it was not possible to estimate the exact frequencies of homozygous individuals (*i.e. valine in both alleles*) or heterozygous individuals (*i.e. leucine in one allele, valine in the other allele*). This is particularly important as most of the pyrethroid mutations are recessive and thus no or little phenotypic effect is expected at the heterozygous state. Nevertheless, a previous study showed high levels of pyrethroid resistance in aphids although the mutation was heterozygous (Panini et al. 2021). In this case, transposon-mediated monoallelic expression prevented the expression of susceptible alleles and thus caused resistant individuals (Panini et al. 2021). Therefore, molecular screening should be combined with phenotypic assays to reveal phenotypic resistance levels.

L925M mutation has been initially reported in the USA and its presence in Europe was documented only recently (González-Cabrera et al. 2016; Millán-Leiva et al. 2021b). A phylogenetic analysis of VGSC haplotypes of *V. destructor* suggested a sequential evolution of these mutations: L925M (CTG → ATG) arose first and then L925I (ATG → ATA) (Millán-Leiva et al. 2021b). However, in this study, we found multiple populations with L925V and L925I mutations without L925M, which was suggested to be the origin of L925I mutation, in line with previous reports from Europe (Alissandrakis et al. 2017; Vlogiannitis et al. 2021b). The presence of a putative fitness cost for the L925M mutation has been suggested, which might explain its absence in cases where L925I is present (Millán-Leiva et al. 2021b). However, there is still a lack of experimental data to support this claim. In addition, we determined the L925M mutation in Turkish *V. destructor* populations, for the first time in Asia, at a prevalence of 14.3%.

Resistance development allows insects to survive exposure to the insecticide, however, it often comes with a trade-off that affects the insect's ability to thrive in other aspects of its life cycle or in its natural environment, a phenomenon referred to as “fitness cost” (Kliot and Ghanim 2012; Ffrench-Constant and Bass 2017). Indeed, a reduction in the number of pyrethroid-resistant *Varroa* mites in the absence of selection pressure has been documented by Milani and Della Vedova (2002). Later on, a putative fitness cost has been associated with L925V/M mutations, based on the correlation observed between the mutation frequency and prior acaricide use (González‑Cabrera et al. 2018; Millán-Leiva et al. 2021b). In the present study, the susceptible leucine at the position 925 was fixed in nine out of 44 samples. Furthermore, it is noteworthy that susceptible genotypes were present in nearly half of the populations. The presence of these susceptible individuals allows for a decrease in resistance levels following cessation of pyrethroid use, primarily due to the associated fitness cost. Thus, alternative control methods or acaricides with different mode of action should be considered in rotation among Turkish bee farmers.

**Fig. 2:**
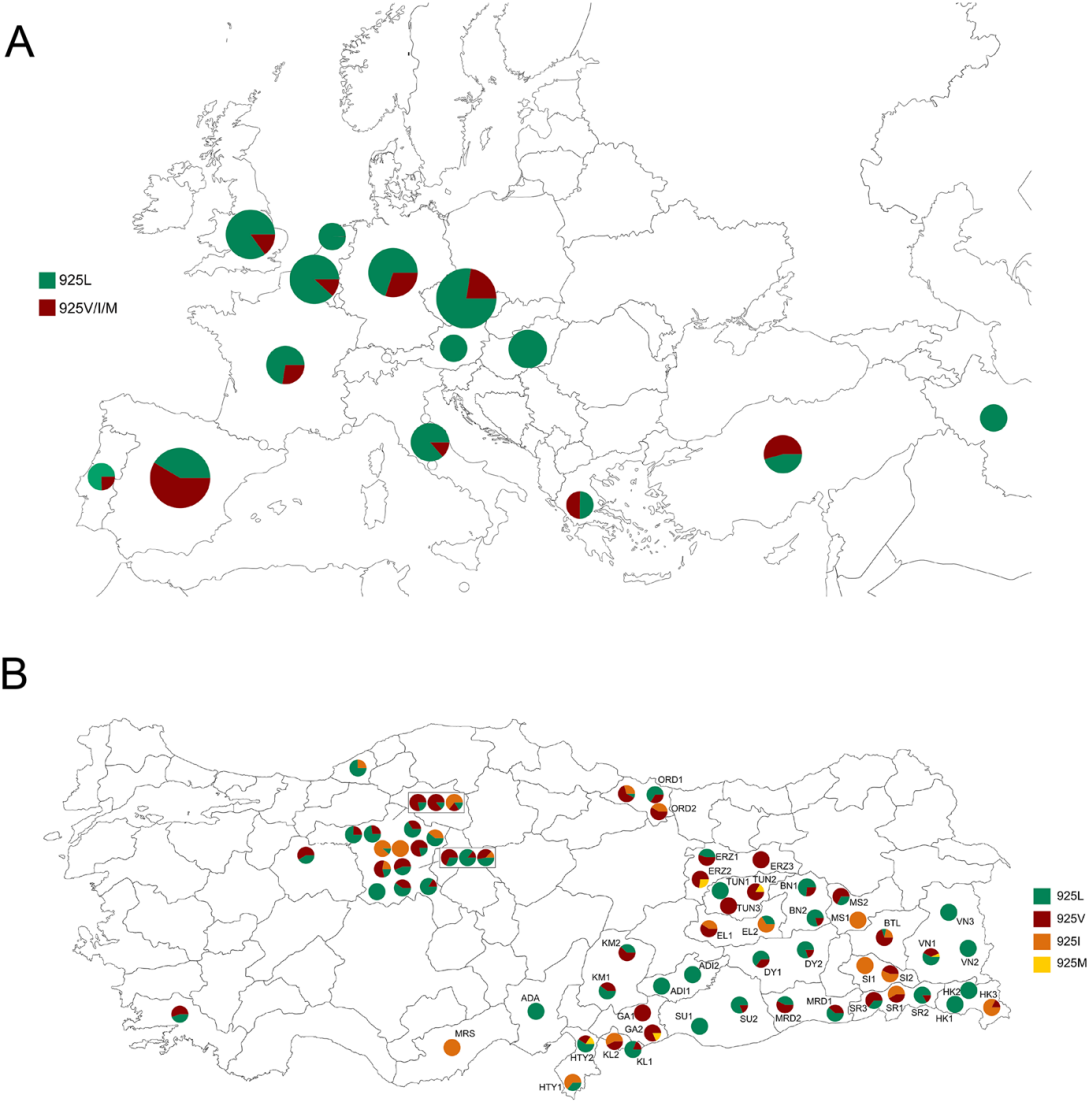
**A** A map showing the distribution of L925V/I/M mutations in Europe *Varroa destructor* populations. Homozygous resistant individuals were represented by red color. Green color were used to show homozygous susceptible and heterezygous individuals (due to recessive nature of mutation). The size of the circles are proportional to the number of samples (1–200; 200–1000; 1000–2500 and > 2500). **B** Spread of L925V/I/M mutations across Türkiye. The samples that were obtained in this study were labeled with their codes on the map (see Table S1 for population codes). The other samples belong to Koç et al. (2021)

In Europe, pyrethroid resistance in *Varroa* mites was initially reported in Italy in the early 1990s (Lodesani et al. 1995). After its first appearance, the resistant individuals quickly spread to neighboring countries such as France, Switzerland, and Slovenia, and subsequently to broader regions across the continent (Martin 2004). Subsequently, the L925V mutation has been identified as the primary driving factor for pyrethroid resistance across Europe, although other variants such as L925I and L925M have also been detected (González-Cabrera et al. 2018). We have compiled the available data to provide an overview of the prevalence and frequency of pyrethroid mutations across Europe (Fig. 2A, Table S3). *V. destructor* populations from Mediterranean countries, namely Türkiye, Spain, and Greece, had the highest frequency of L925V/M/I mutations (Fig. 2A). While pyrethroid usage is not widespread throughout Europe, the percentage of beekeepers in Spain and Greece employing tau-fluvalinate and flumethrin, respectively, is higher than in most other European countries (Brodschneider et al. 2023). Likewise, flumethrin has been extensively employed in Turkish apiaries (Koç et al. 2021; this study). The more frequent use of pyrethroids in these countries has likely contributed to the widespread presence of pyrethroid mutations. Additionally, the substantially faster population growth of *Varroa* mites in Mediterranean climates, compared to temperate regions, is likely caused by the consistent presence of brood, potentially contributing to resistance development (Kraus and Page Jr 1995). Hence, it is advisable to urgently prioritize alternative control methods, especially in Mediterranean countries with a high mutation frequency. On the other hand, none of the *Varroa* samples from Hungary, Austria, and Iran and only 1 out of 172 samples from the Netherlands had L925 mutations, indicating low pyrethroid selection pressure in these areas. The dominance of susceptible mites (homozygous susceptible and heterozygous) across Europe except in southern parts indicates the rational use of pyrethroids. Indeed, control practices in Europe have demonstrated the utilization of alternative control measures (Brodschneider et al. 2023). Nevertheless, the presence of pyrethroid resistant individuals must be considered during the design of *Varroa* management programs, as their frequency can be rapidly increased in case of repetitive use.

### Supplementary Information

Below is the link to the electronic supplementary material.

Supplementary file1 (PDF 39 KB)

Supplementary file1 (DOCX 26 KB)
