# Supplementary figures and images for: Geographical distribution of pyrethroid resistance mutations in Varroa destructor across Türkiye and a European overview
Source: Exp Appl Acarol. 2024 Feb 24;92(3):309–21. doi: 10.1007/s10493-023-00879-z (PMC11035437; doi:10.1007/s10493-023-00879-z)

# VdAChE1

*V. destructor* numbering  
(*T. californica* numbering)

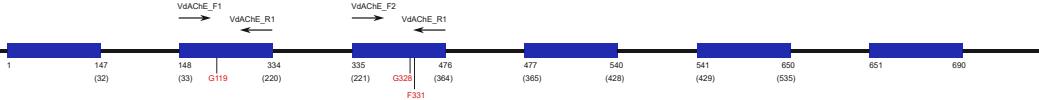

Supplement: Supplementary file 1 — Supplementary file1 (PDF 39 KB) [file 10493_2023_879_MOESM1_ESM.pdf]
