# Supplementary material for: Geographical distribution of pyrethroid resistance mutations in Varroa destructor across Türkiye and a European overview
Source: Exp Appl Acarol. 2024 Feb 24;92(3):309–21. doi: 10.1007/s10493-023-00879-z (PMC11035437; doi:10.1007/s10493-023-00879-z)
Supplement: Supplementary file 2 — Supplementary file1 (DOCX 26 KB) [file 10493_2023_879_MOESM2_ESM.docx]

*Fig. S1. Gene structure and the fragments amplified to screen mutations at positions previously associated with resistance to AChE inhibitors. Blue rectangles indicate exons, while black lines indicate introns. The numbering is according to Varroa destructor AChE (XP_022645444) and Torpedo californica when they are used in paranthesis. The previously reported mutations located within amplification sites are indicated in red font according to T. californica numbering.*

Table S1. Locations, beekeeping type, insecticide usage history, collection and last application dates of sampled *Varroa destructor* populations

| **Population (code)** | **Location** | **Beekeeping Type** | **Insecticide usage history** | **Collection Date** | **Last application** |
| --- | --- | --- | --- | --- | --- |
| ADA | Adana/Ceyhan | [Migratory](https://tureng.com/tr/turkce-ingilizce/migratory%20beekeeping) | Amitraz | June 2022 | Spring 2022 |
| ADI1 | Adıyaman/Kahta | [Migratory](https://tureng.com/tr/turkce-ingilizce/migratory%20beekeeping) | Flumethrin, amitraz | June 2022 | 3-4 days ago |
| ADI2 | Adıyaman/Kahta | [Migratory](https://tureng.com/tr/turkce-ingilizce/migratory%20beekeeping) | Flumethrin, amitraz, organic acids | June 2022 | 3-4 days ago |
| BN1 | Bingöl/Centrum | Stationary | Amitraz, essential oils, organic acids | June 2022 | Unknown |
| BN2 | Bingöl/Centrum | Stationary | Flumethrin, organic acids | June 2022 | Unknown |
| BTL | Bitlis/Hizan | Stationary | Amitraz, organic acids | June 2022 | Spring 2022 |
| DY1 | Diyarbakır/Kayapınar | [Migratory](https://tureng.com/tr/turkce-ingilizce/migratory%20beekeeping) | Flumethrin, amitraz | June 2022 | Autumn 2021 |
| DY2 | Diyarbakır/Çınar | [Migratory](https://tureng.com/tr/turkce-ingilizce/migratory%20beekeeping) | Flumethrin, amitraz | June 2022 | Autumn 2021 |
| EL1 | Elazığ/Kovancılar | [Migratory](https://tureng.com/tr/turkce-ingilizce/migratory%20beekeeping) | Amitraz, organic acids | June 2022 | 20-25 days ago |
| EL2 | Elazığ/Karakorçan | Stationary | Flumethrin, amitraz, organic acids, essential oils | June 2022 | 10-15 days ago |
| ERZ1 | Erzincan/Ergandağı | [Stationary](https://tureng.com/tr/turkce-ingilizce/migratory%20beekeeping) | Amitraz, flumethrin, organic acids, essential oils | June 2022 | Autumn 2021 |
| ERZ2 | Erzincan/Yaylabaşı | [Migratory](https://tureng.com/tr/turkce-ingilizce/migratory%20beekeeping) | Amitraz, flumethrin, organic acids, essential oils | June 2022 | Autumn 2021 |
| ERZ3 | Erzincan/Tercan | Stationary | Amitraz, organic acids | June 2022 | Autumn 2021 |
| GA1 | Gaziantep/Nizip | Stationary | Amitraz | June 2022 | 20-25 days ago |
| GA2 | Gaziantep/Nizip | Stationary | Amitraz | June 2022 | 20-25 days ago |
| HK1 | Hakkari/Yüksekova | Stationary | Flumethrin, amitraz, organic acids | June 2022 | 20 days ago |
| HK2 | Hakkari/Yüksekova | Stationary | - | June 2022 | Unknown |
| HK3 | Hakkari/Yüksekova | Stationary | Amitraz | June 2022 | 20 days ago |
| HTY1 | Hatay/Dörtyol | [Migratory](https://tureng.com/tr/turkce-ingilizce/migratory%20beekeeping) | Flumethrin, amitraz, | June 2022 | 45 days ago |
| HTY2 | Hatay/Erzin | [Migratory](https://tureng.com/tr/turkce-ingilizce/migratory%20beekeeping) | Amitraz | June 2022 | 15-20 days ago |
| KM1 | Kahramanmaraş/Pazarcık | Stationary | Amitraz, essential oils | June 2022 | 15-20 days ago |
| KM2 | Kahramanmaraş/Pazarcık | Stationary | Amitraz, essential oils | June 2022 | 15-20 days ago |
| KL1 | Kilis/Centrum | Stationary | Amitraz | June 2022 | 2 weeks ago |
| KL2 | Kilis/Centrum | Stationary | Amitraz | June 2022 | 15-20 days ago |
| MRD1 | Mardin/Mazıdağı | [Migratory](https://tureng.com/tr/turkce-ingilizce/migratory%20beekeeping) | Amitraz | June 2022 | Spring 2022 |
| MRD2 | Mardin/Mazıdağı | [Migratory](https://tureng.com/tr/turkce-ingilizce/migratory%20beekeeping) | Amitraz | June 2022 | Spring 2022 |
| MRS | Mersin/Centrum | [Migratory](https://tureng.com/tr/turkce-ingilizce/migratory%20beekeeping) | Amitraz, essential oils | June 2022 | 15-20 days ago |
| MS1 | Muş/Karlıdere | [Stationary](https://tureng.com/tr/turkce-ingilizce/migratory%20beekeeping) | Amitraz, essential oils | June 2022 | 15 days ago |
| MS2 | Muş/Karlıdere | [Stationary](https://tureng.com/tr/turkce-ingilizce/migratory%20beekeeping) | Amitraz | June 2022 | 3-4 days ago |
| ORD1 | Ordu/Centrum | [Migratory](https://tureng.com/tr/turkce-ingilizce/migratory%20beekeeping) | Flumethrin, amitraz | June 2022 | 15-20 days ago |
| ORD2 | Ordu/Centrum | [Migratory](https://tureng.com/tr/turkce-ingilizce/migratory%20beekeeping) | Amitraz, organic acids, essential oils | June 2022 | 15-20 days ago |
| SI1 | Siirt/Pervari | Stationary | Essential oils | June 2022 | Autumn 2021 |
| SI2 | Siirt/Şirvan | Stationary | Amitraz | June 2022 | Spring 2022 |
| SU1 | Şanlıurfa/Karacadağ | [Migratory](https://tureng.com/tr/turkce-ingilizce/migratory%20beekeeping) | Amitraz, organic acids | June 2022 | 20 days ago |
| SU2 | Şanlıurfa/Ceylanpınar | [Migratory](https://tureng.com/tr/turkce-ingilizce/migratory%20beekeeping) | Amitraz, organic acids, essential oils | June 2022 | 15-20 days ago |
| SR1 | Şırnak/Silopi | [Migratory](https://tureng.com/tr/turkce-ingilizce/migratory%20beekeeping) | Amitraz | May 2022 | Autumn 2021 |
| SR2 | Şırnak/Cizre | [Migratory](https://tureng.com/tr/turkce-ingilizce/migratory%20beekeeping) | Amitraz, organic acids | May 2022 | 3-4 days ago |
| SR3 | Şırnak/İdil | Stationary | Organic acids | April 2022 | Autumn 2021 |
| TUN1 | Tunceli/Ovacık | [Migratory](https://tureng.com/tr/turkce-ingilizce/migratory%20beekeeping) | Amitraz, flumethrin, organic acids, essential oils | June 2022 | 15-20 days ago |
| TUN2 | Tunceli/Ovacık | Stationary | Amitraz, organic acids, essential oils | June 2022 | 3-4 days ago |
| TUN3 | Tunceli/Pülümür | Stationary | Amitraz, flumethrin, organic acids, essential oils | June 2022 | 15-20 days ago |
| VN1 | Van/Gevaş | Stationary | Amitraz | June 2022 | 30 days ago |
| VN2 | Van/Gevaş | [Migratory](https://tureng.com/tr/turkce-ingilizce/migratory%20beekeeping) | Amitraz | June 2022 | Spring 2022 |
| VN3 | Van/Başkale | Stationary | - | June 2022 | Unknown |

Table S2. Presence of mutations, their frequency, accession numbers of *COI* and *ND4* sequences of sampled *Varroa destructor* populations

| **Population** | **Allele^1^** | **Allele frequency^2^** | | | | **Accession numbers** | |
| --- | --- | --- | --- | --- | --- | --- | --- |
|  |  | **L** | **V** | **M** | **I** | **COI** | **ND4** |
| ADA | L | 100 | - | - | - | OR946023 | OR964206 |
| ADI1 | L | 100 | - | - | - | OR946024 | OR964207 |
| ADI2 | L | 100 | - | - | - | OR946025 | OR964208 |
| BN1 | L/V | 73.4 | 26.6 | - | - | OR946026 | OR964209 |
| BN2 | L/V | 79.8 | 20.2 | - |  | OR946027 | OR964210 |
| BTL | V/I/L | 8.2 | 70 | - | 21.8 | OR946028 | OR964211 |
| DY1 | L/V | 64.7 | 35.3 | - |  | OR946029 | OR964212 |
| DY2 | L/V | 80.1 | 19.9 | - |  | OR946030 | OR964213 |
| EL1 | V/I | - | 59 | - | 41 | OR946031 | OR964214 |
| EL2 | I/L | 33.7 |  | - | 66.3 | OR946032 | OR964215 |
| ERZ1 | V/L | 45.2 | 54.8 | - | **-** | OR946033 | OR964216 |
| ERZ2 | V/M | - | 72.9 | 27.1 | **-** | OR946034 | OR964217 |
| ERZ3 | V | - | 100 |  | **-** | OR946035 | OR964218 |
| GA1 | V | - | 100 |  | - | OR946036 | OR964219 |
| GA2 | V/M | - | 81.9 | 18.1 | - | OR946037 | OR964220 |
| HK1 | L | 100 | - | - | - | OR946038 | OR964221 |
| HK2 | L | 100 | - | - | - | OR946039 | OR964222 |
| HK3 | I/V | - | 17.7 | - | 82.3 | OR946040 | OR964223 |
| HTY1 | I/L | 35.7 | - | - | 64.3 | OR946041 | OR964224 |
| HTY2 | L/V/M | 59.1 | 25.2 | 15.7 | - | OR946042 | OR964225 |
| KM1 | L/V | 60 | 40 | - | - | OR946043 | OR964226 |
| KM2 | V/L | 38 | 62 | - | - | OR946044 | OR964227 |
| KL1 | L/V | 81.3 | 18.7 | - | - | OR946045 | OR964228 |
| KL2 | I/V | - | 42.9 | - | 57.1 | OR946046 | OR964229 |
| MRD1 | L/V | 61.7 | 38.3 | - | - | OR946047 | OR964230 |
| MRD2 | V/L | 42.8 | 57.2 | - | - | OR946048 | OR964231 |
| MRS | I | - | - | - | 100 | OR946049 | OR964232 |
| MS1 | I | - | - | - | 100 | OR946050 | OR964233 |
| MS2 | V/L | 32.6 | 67.4 | - | - | OR946051 | OR964234 |
| ORD1 | L/V | 66.1 | 33.9 | - | - | OR946052 | OR964235 |
| ORD2 | V/I | - | 58.6 | - | 41.4 | OR946053 | OR964236 |
| SI1 | I | - | - | - | 100 | OR946054 | OR964237 |
| SI2 | I/V | - | 43.6 | - | 56.4 | OR946055 | OR964238 |
| SU1 | L | 100 | - | - | - | OR946056 | OR964239 |
| SU2 | L/V | 81.5 | 18.5 | - | - | OR946057 | OR964240 |
| SR1 | I/V | - | 43.6 | - | 56.4 | OR946058 | OR964241 |
| SR2 | L/V | 83.4 | 16.6 | - | - | OR946059 | OR964242 |
| SR3 | V/L | 35.4 | 64.6 | - | - | OR946060 | OR964243 |
| TUN1 | L | 100 | - | - | - | OR946061 | OR964244 |
| TUN2 | V | - | 100 | - | - | OR946062 | OR964245 |
| TUN3 | V/M | - | 83.3 | 16.7 | - | OR946063 | OR964246 |
| VN1 | L/V/M | 57.4 | 36.4 | 6.2 | - | OR946064 | OR964247 |
| VN2 | L | 100 | - | - | - | OR946065 | OR964248 |
| VN3 | L | 100 | - | - | - | OR946066 | OR964249 |

^1^ Amino acids are presented in decreasing order of estimated frequency

^2^ Allele frequencies were determined according to Van Leeuwen et al. (2008)

Table S3. Details on the data used to create the map and references

| **Country** | **n** | **SS/SR** | **RR** | **References** |
| --- | --- | --- | --- | --- |
| UK | 1776 | 1509 | 267 | González‑Cabrera et al. 2013; González -Cabrera et al. 2018 |
| Greece | 113 | 57 | 56 | Alissandrakis et al. 2017 |
| Czechia | 5581 | 4322 | 1259 | Hubert et al. 2014; Stara et al. 2019 |
| Austria | 48 | 48 | 0 | González‑Cabrera et al. 2018 |
| Belgium | 1116 | 984 | 132 | González‑Cabrera et al. 2018  Vlogiannitis et al. 2021 |
| France | 971 | 703 | 268 | González‑Cabrera et al. 2018; Almecija et al. 2022 |
| Germany | 1102 | 770 | 332 | González‑Cabrera et al. 2018 |
| Hungary | 542 | 542 | 0 | González‑Cabrera et al. 2018 |
| Italy | 671 | 580 | 91 | González‑Cabrera et al. 2018; Panini et al. 2019 |
| Spain | 6402 | 2656,3 | 3745,7 | González‑Cabrera et al. 2018; Hernández-Rodríguez et al. 2021; Benito-Murcia et al. 2022 |
| The Netherlands | 172 | 171 | 1 | González‑Cabrera et al. 2018 |
| Portugal | 192 | 145 | 47 | Li et al. 2022 |
| Iran | 28 | 28 | 0 | Farjamfar et al. 2018 |
| Türkiye | 660 | 302 | 358 | Koç et al., 2021; This study |

**References for Table S3**

Alissandrakis E, Ilias A, Tsagkarakou A (2017) Pyrethroid target site resistance in Greek populations of the honey bee parasite Varroa destructor (Acari: Varroidae). J Apic Res 56(5):625–630

Almecija G, Schimmerling M, Del Cont A, Poirot B, Duquesne V (2022) Varroa destructor resistance to tau‐fluvalinate: relationship between in vitro phenotypic test and VGSC L925V mutation. Pest Manag Sci 78(12):5097-5105.

Benito-Murcia M, Martín-Hernández R, Meana A, Botías C, Higes M (2022) Study of pyrethroid resistance mutations in populations of Varroa destructor across Spain. Res Vet Sci 152:34-37.

Farjamfar M, Saboori A, González-Cabrera J, Rodríguez CSH (2018) Genetic variability and pyrethroid susceptibility of the parasitic honey bee mite Varroa destructor (Acari: Varroidae) in Iran. Exp Appl Acarol 76(1):139–148

González-Cabrera J, Bumann H, Rodríguez-Vargas S, Kennedy PJ, Krieger K, Altreuther G, Hertel A, Hertlein G, Nauen R, Williamson MS (2018) A single mutation is driving resistance to pyrethroids in European populations of the parasitic mite, Varroa destructor. J Pest Sci 91(3):1137–1144

González-Cabrera J, Davies TE, Field LM, Kennedy PJ, Williamson MS (2013) An amino acid substitution(L925V) associated with resistance to pyrethroids in Varroa destructor. PLoS One 8(12):e82941

Hernández-Rodríguez CS, Marín Ó, Calatayud F, Mahiques MJ, Mompó A, Segura I, Simó E, González-Cabrera J (2021) Large-scale monitoring of resistance to coumaphos, amitraz, and pyrethroids in *Varroa destructor*. Insects 12(1):27

Hubert J, Nesvorna M, Kamler M, Kopecky J, Tyl J, Titera D, Stara J (2014) Point mutations in the sodium channel gene conferring tau-fluvalinate resistance in Varroa destructor. Pest Manag Sci 70(6):889–894

Koç N, İnak E, Jonckheere W, Van Leeuwen T (2021) Genetic analysis and screening of pyrethroid resistance mutations in Varroa destructor populations from Turkey. Exp Appl Acarol 84(2):433-444.

Li F, Teixeira J, Ferreira HM, Valério MJ, Russo-Almeida PA (2022) Varroa destructor in Portugal: an exploratory assessment of pyrethroids resistance status. Journal Apic Res 1-4.

Panini M, Reguzzi MC, Chiesa O, Cominelli F, Lupi D, Moores G, Mazzoni E (2019) Pyrethroid resistance in Italian populations of the mite Varroa destructor: a focus on the Lombardy region. Bull Insectol 72(2):227–232

Stara J, Pekar S, Nesvorna M, Kamler M, Doskocil I, Hubert J (2019) Spatio-temporal dynamics of Varroa destructor resistance to tau-fluvalinate in Czechia, associated with L925V sodium channel point mutation. Pest Manag Sci 75(5):1287–1294

Vlogiannitis S, Jonckheere W, Laget D, de Graaf DC, Vontas J, Van Leeuwen T. (2021). Pyrethroid target-site resistance mutations in populations of the honey bee parasite Varroa destructor (Acari: Varroidae) from Flanders, Belgium. Exp Appl Acarol 85(2-4): 205-221.

Table S4. COI haplotypes of *Varroa destructor* populations across the globe

| Haplotype | n | Populations/accession numbers |
| --- | --- | --- |
| Hap_1 | 241 | ADA ADI1 ADI2 GA1 GA2 BN1 ORD1 ORD2 SI2 SR1 SR2 SR3 SU2 VN1 VN2 VN3 BN2 BTL TUN1 TUN2 TUN3 DY1 DY2 HK1 KL1 KL2 HK2 HK3 HTY1 KM1 SI1 SU1 MRD1 HTY2 KM2 MRD2 MRS MS1 MS2 EL1 EL2 ERZ1 ERZ2 ERZ3 GQ379056.1 AP019523.1 AJ493124.2 GQ379057.1 GQ379058.1 GQ379060.1 JX970939.1 MG793455.1 MT462459.1 MT462460.1 MT462461.1 MT462462.1 MT462463.1 MT462464.1 MT462465.1 MT462466.1 MT462467.1 MT462468.1 NC_004454.2 JX970938.1 MW725322.1 MW599128.1 MW599129.1 MW599130.1 MW599140.1 MW599141.1 MW599145.1 MW599146.1 MW599147.1 KR528381.1 KR528384.1 MW725321.1 MW725319.1 KR528385.1 MW725307.1 MW725308.1 MW725318.1 MW725315.1 MW725320.1 OK560013.1 OK560014.1 OK560015.1 OK560016.1 OM929188.1 OM929189.1 OM929190.1 MW725309.1 MW725310.1 MW725312.1 MW725311.1 MW725313.1 MW725314.1 MW725316.1 MW725317.1 OP420871.1 OP420872.1 OP420873.1 OP420874.1 OP420876.1 OP420878.1 OP420879.1 OP420882.1 OP420883.1 OP420884.1 OP420885.1 OP420886.1 OP420887.1 OP420888.1 OP420889.1 OP420890.1 OP420891.1 OP420893.1 OP420894.1 OP420896.1 OP420897.1 OP420898.1 OP420899.1 OP420900.1 OP420901.1 OP420902.1 OP420903.1 OP420904.1 OP420905.1 OP420906.1 OP420908.1 OP420909.1 OP420911.1 OP420912.1 OP420913.1 OP420914.1 OP420915.1 OP420916.1 OP420917.1 OP420918.1 OP420919.1 OP420920.1 OP420921.1 OP420922.1 OP420923.1 OP420924.1 OP420925.1 OP420926.1 OP420927.1 OP420928.1 OP420929.1 OP420930.1 OP420931.1 OP420932.1 OP420933.1 OP420934.1 OP420935.1 OP420936.1 OP420937.1 OP420938.1 OP420939.1 OP420940.1 OP420941.1 OP420942.1 OP420943.1 OP420944.1 OP420945.1 OP420946.1 OP420947.1 OP420948.1 OP420949.1 OP420950.1 OP420951.1 OP420952.1 OP420953.1 OP420954.1 OP420955.1 OP420956.1 OP420957.1 OP420958.1 OP420959.1 OP420960.1 OP420961.1 OP420962.1 OP420963.1 OP420964.1 OP420966.1 OP420967.1 OP420968.1 OP420969.1 OP420970.1 OP420971.1 OP420972.1 OP420875.1 OP420907.1 OP420965.1 OK626252.1 OK626253.1 OK626254.1 OK626255.1 OK626256.1 OK626257.1 OK626258.1 OK626259.1 OK626260.1 LN873226.1 OP984055.1 OP984078.1 OQ254749.1 OQ410463.1 MN360198.1 MN362120.1 OQ205274.1 OQ205275.1 OQ205276.1 OQ205277.1 OQ205279.1 OQ205281.1 OQ205282.1 KY865176.1 KY865177.1 KY865178.1 KY865179.1 KY865180.1 KY865181.1 KY865182.1 KY865183.1 KY865184.1 KY865185.1 MW504309.1 MW504310.1 MW504311.1 MW504312.1 MW504313.1 MW504314.1 MW504315.1 MW504316.1 MW504317.1 OK063784.1 OK142785.1 ON901872.1 ON901873.1 MN364121.1 |
| Hap_2 | 1 | LN873222.1 |
| Hap_3 | 21 | GQ379059.1 GQ379069.1 GQ379070.1 GQ379071.1 GQ379072.1 GQ379073.1 GQ379074.1 AJ784872.1 MW599107.1 MW599108.1 MW599109.1 MW599110.1 MW599111.1 MW599112.1 MW599113.1 MW599114.1 MW599115.1 MW599116.1 MW599117.1 MW599118.1 MW599119.1 |
| Hap_4 | 2 | KR528386.1 KR528387.1 |
| Hap_5 | 3 | MW599134.1 MW599138.1 MW599139.1 |
| Hap_6 | 1 | OQ205284.1 |
| Hap_7 | 2 | GQ379067.1 GQ379068.1 |
| Hap_8 | 14 | GQ379061.1 GQ379062.1 GQ379063.1 KR528382.1 MW599122.1 MW599123.1 MW599127.1 MW599137.1 MW599148.1 MW599149.1 MW599125.1 MW599142.1 MW599143.1 MW599144.1 |
| Hap_9 | 23 | GQ379064.1 GQ379065.1 GQ379066.1 KR528378.1 KR528379.1 KR528380.1 KR528383.1 MW599120.1 MW599121.1 MW599124.1 MW599131.1 MW599132.1 MW599133.1 MW599135.1 MW599136.1 MW599150.1 MW599151.1 MW599152.1 MW599153.1 MW599154.1 MW599155.1 MW599156.1 MW599126.1 |
| Hap_10 | 5 | OP420877.1 OP420895.1 OP420910.1 OP420881.1 OP420892.1 |
| Hap_11 | 2 | OQ205278.1 OQ205280.1 |
| Hap_12 | 1 | ON901876.1 |
| Hap_13 | 1 | ON901874.1 |
| Hap_14 | 1 | ON901875.1 |
| Hap_15 | 1 | ON901877.1 |
| Hap_16 | 1 | MK509767.1 |
| Hap_17 | 1 | OQ205283.1 |
| Hap_18 | 1 | MN175996.1 |
